# Molecular Characterization and Mineralizing Potential of Phosphorus Solubilizing Bacteria Colonizing Common Bean (*Phaseolus vulgaris* L.) Rhizosphere in Western Kenya

**DOI:** 10.1155/2023/6668097

**Published:** 2023-03-01

**Authors:** Kelvin Kiprotich, John Muoma, Dennis O. Omayio, Tavasi S. Ndombi, Clabe Wekesa

**Affiliations:** Department of Biological Sciences, School of Natural Sciences, Masinde Muliro University of Science and Technology, P.O. Box 190, Kakamega 50100, Kenya

## Abstract

Phosphorus solubilizing bacteria (PSB) are a category of microbes that transform insoluble phosphates in soil into soluble forms that crops can utilize. Phosphorus in natural soils is abundant but poorly soluble. Hence, introducing PSB is a safer way of improving its solubility. The aim of this study was to genetically characterize and determine the mineralization capability of selected PSB colonizing rhizospheres of common beans in Western Kenya. Seven potential phosphorus solubilizing bacteria (PSB) were isolated from various subregions of Western Kenya. 16S ribosomal RNA gene sequencing and National Center for Biotechnology Information (NCBI), Basic Local Alignment Search Tool (BLAST) identified the isolates. The phosphate solubilization potential of the isolates was evaluated under agar and broth medium of National Botanical Research Institute's phosphate (NBRIP) supplemented with tricalcium calcium phosphate (TCP). Identified isolates were as follows: KK3 as *Enterobacter mori*, B5 (KB5) as *Pseudomonas kribbensis*, KV1 as *Enterobacter asburiae*, KB3 as *Enterobacter mori,* KK1 as *Enterobacter cloacae*, KBU as *Enterobacter tabaci*, and KB2 as *Enterobacter bugandensis*. The strains B5 and KV1 were the most effective phosphorus solubilizers with 4.16 and 3.64 indices, respectively. The microbes converted total soluble phosphate concentration in broth medium which was 1395 and 1471 P *μ*g/mL, respectively. The least performing isolate was KBU with a 2.34 solubility index. Significant (*p* ≤ 0.05) differences in plant biomass for Rose coco and Mwitemania bean varieties were observed under inoculation with isolates B5 and KV1. PSB isolates found in common bean rhizospheres exhibited molecular variations and isolates B5 and KV1 are the potential in solving the insufficiency of phosphorus for sustainable crop production.

## 1. Introduction

Phosphorus (P) is the second most important nutrient for plant growth and development. It plays a significant role in key metabolic pathways such as nutrient uptake, biological oxidation, and energy metabolism [1]. Crops need significant nutrients in order to grow and produce substantial yields in any production system [2, 3]. The urgent need to feed the world's ever-growing population is putting immense strain on arable land around the world [4]. The quality of food-producing habitats have depreciated overtime due to land overuse and excessive application of destructive inorganic fertilizers [5]. Nitrogen, phosphorus, and potassium (NPK) fertilizers have been widely used in agricultural practice around the world to provide macronutrients that promote plant growth and, as an outcome, increase crop productivity [6]. Chemical fertilizers have undoubtedly provided benefits to modern cropping systems, but their overuse has harmed the health of agricultural soils and disrupted the important plant growth-promoting rhizobacteria (PGPR), resulting in lower production [7]. Due to environmental and health concerns brought up by the pervasive usage of chemical fertilizers to deliver nutrients in agriculture [8], current studies are focusing on developing alternative technologies to reduce reliance on chemical phosphate fertilizers and facilitate the widespread use of bioinoculants in agronomic practices [9].

The modern application of microorganisms that support plant growth and development includes the inoculation of rhizobacteria and mycorrhizae [10]. Phosphorus solubilizing bacteria (PSB) are among well-known rhizobacteria that enrich plant growth characteristics [11]. The vast majority of these soil bacteria are among *Pseudomonas* spp., *Enterobacter* spp., *Agrobacterium* spp., and *Bacillus* spp. that have been testified to activate poorly available phosphorus through solubilization and mineralization [12–14]. These microorganisms have been identified as having a high potential for phosphorus solubilization [15]. In soil and plant rhizospheres, multiple phosphorous solubilizing bacteria have been discovered, each with its own different ability to solubilize phosphates [16]. The solubilization potential of these bacteria, on the other hand, varies genetically, ecologically, and by plant type [17]. Assessment of potential phosphorus solubilizing bacteria for specific regions that can be used as bioinoculants/biofertilizers to boost plant growth performance and improve yields is considered as an emerging and sustainable field because such bacterial inoculants can credibly moderate the disproportionate use of chemical fertilizers while also preserving soil microflora [4, 18].

The 16S ribosomal RNA gene is a conserved gene across all prokaryotes but with hypervariable regions has been used to genetically characterize phosphorus-solubilizing bacteria all over the world [17, 19, 20] but less has been carried out in Kenya for plant-promoting microorganisms, particularly in crop production zones of Western Kenya, Rift Valley, and Central Kenya. The purpose of this study was to isolate and genetically characterize phosphorous solubilizing bacteria, as well as determine their phenotypic effects on the growth and development of common beans. Furthermore, the study sought to determine the levels of phosphate solubilization in broth and agar medium. Determining potential PSB isolates associated with common beans in Western Kenya, besides comparing and analyzing their phylogenetic relationship and mineralization potential, would be a major step in developing efficient bioinoculants for safer, economically sustainable agricultural systems that protects the soil from hazardous chemical fertilizers [21].

## 2. Methodology

### 2.1. Study Region

**Figure 1:**
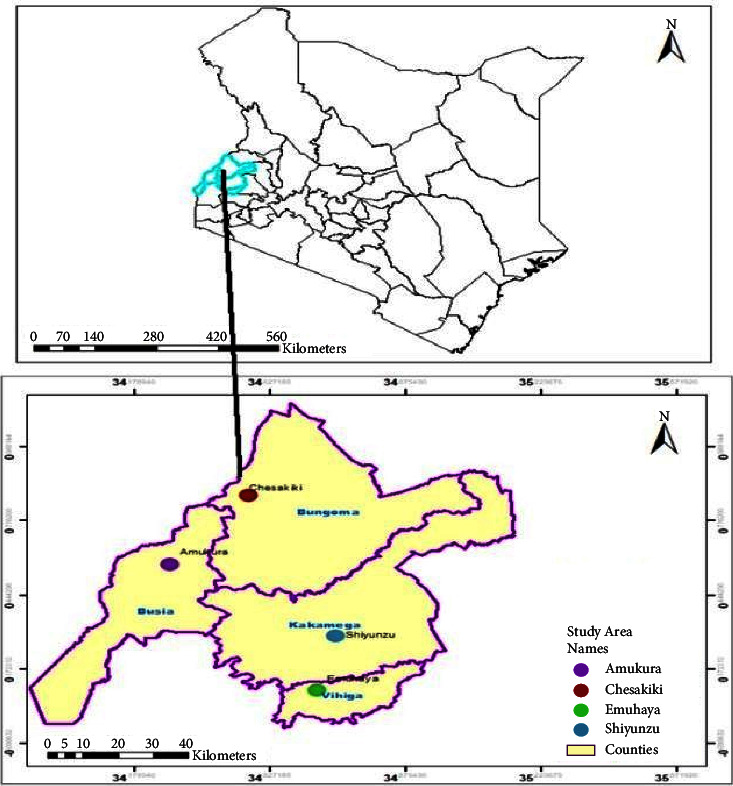
Study area map.

The sites of isolation that represented Western Kenya were selected based on centric random systematic sampling from respective counties and subcounties (Figure 1). They were Lurambi (N 0° 0.29“; E 34° 69.71′) in Kakamega County, Emuhaya (N 0° 5.42′; E 34° 34.65′) in Vihiga County, Teso South (N 0° 33.729′; E 34° 16.21′) in Busia County, and Chaptais (N 0° 48.36′; E 34° 28.26′) in Bungoma County. Samples were collected during mid of June 2021. The main source of income of Western Kenya inhabitants is mixed agricultural farming [22]. Sugarcane, maize, beans, finger millets, bananas, and sweet potatoes are among the main food and cash crops grown in the region [23]. Western Kenya is typically hot and humid, with year-round rainfall. According to the World Bank Climate Change Knowledge Portal, it is indicated that it received average temperature of 21.28°C and an average rainfall of 2233.59 mm in the year 2021.

### 2.2. Bacteria Isolation

Root nodules and rhizosphere soil surrounding uprooted common bean were used to isolate bacteria using the method described by Tomer et al. [24]. Briefly, flowered bean plants were uprooted with a portion of the soil, and the root nodules were collected into sterilized khaki paper bags and taken to the laboratory for morphological identification of phosphorus solubilizing bacteria within 24 hours. Homogenate of root nodules and rhizosphere soil (10% soil in 0.85% saline water) was made using a mortar and pestle followed by serial dilutions which were prepared within 24 hours at room temperature according to Pande et al. [25]. A droplet of liquid in diluents in the test tubes was placed on the midpoint of the sterile agar plate and uniformly spread across the surface with the help of a sterilized glass-rod and incubated for five days at 28°C. Subculturing was carried out to obtain the pure isolates [26].

### 2.3. Phosphorus Solubilizing Bacteria Isolation

Isolates were grown on both solid and liquid nutrient medium of the National Botanical Research Institute's Phosphate Growth Medium (NBRIP) supplemented with tricalcium phosphate [27]. NBRIP contains 10 grams of glucose substrate, 5 grams of Ca_3_(PO_4_)_2_, 5 grams of MgCl_2_.6H_2_O, 0.25 grams of MgSO_4_.7H_2_O, 0.2 grams of KCl, 0.1 grams of (NH_4_)_2_SO_4_, and 15 grams of agar in 1000 milliliters of distilled water. The pH of the media was maintained at 7.0 before autoclaving. Bacterial strains were introduced into the media by the standard pour plate technique using a sterile dropper (10 *μ*L of aliquots per plate) [28]. They were incubated for 7 days at 28 °C. At the end of the incubation, PSB were able to grow and were identified through the formation of a halo zone around the colony [29]. Colonies that did not form the halo zone were exempted. The colony diameter (C.D) and halo zone diameter (H.D) of each isolate were measured and the solubilizing index (SI) was calculated. Seven efficient PSB isolates for further experiments were selected based on maximum phosphorus solubility potential.

The phosphorous solubilization index of the isolates was determined by the following equation [30]:

(1) $$\begin{array}{c} Solubilizing\;Index\left(SI\right)=\frac{Isolate^{\prime }s\;Colony\;Diameter\left(C.D\right)+Isolate^{\prime }s\;Halo\;Zone\left(H.D\right.}{Isolate^{\prime }s\;Colony\;Diameter\left(C.D\right)}\text{.} \end{array}$$

### 2.4. Determinations of Amount of Solubilized Phosphate

A culture of 1 mL of the isolated strains (OD_600_ = 0.5 nm) was inoculated separately into 250 ml conical flask containing 150 mL of liquid NBRIP medium supplemented with 0.5% tricalcium phosphate (Thomas Baker, Mumbai India) and incubated at 28°C for 24 hours. Sterile water inoculated into a medium was treated as a control. Approximately 1 mL of the supernatant was used after 18000 × *g* centrifugation for 5-minute to assess phosphorus released into the solution. Phosphorus in the supernatant was determined by the molybdenum blue colorimetric method according to Murphy and Riley [31]. The reagents were made up of ascorbic acid and antimony containing acidified ammonium molybdate solution. This substance combines quickly with the phosphate ion to produce a blue-purple molecule that has an atomic ratio of 1 : 1 antimony to phosphorus. As long as there is at least 2 g/mL of phosphate in the solution, the complex is extremely stable and follows Beer's law. The absorbance was measured at a wavelength of 800 nm with a ultraviolet and visible range spectrophotometer.

### 2.5. Determination of Phosphatase Enzyme Activity

Phosphatase activity was estimated according to Behera et al. [32] protocol. Microbial culture (1.5 mL) of 24 hr actively growing PSB culture inoculated in a 250 ml of NBRIP broth was pipetted into 2 ml Eppendorf tube and centrifuged at 12000 × *g* for 10 min at 4°C. Solution (4 ml) of modified universal buffer (MUB) (pH 6.5) was mixed with 1 mL culture supernatant followed by addition of 1 mL of 0.115 M disodium *p-*nitrophenyl phosphate (tetrahydrate) and incubated at 37°C for 1 hr. Drops of Toluene were added to the mixture to end the growth of the microbial culture. After incubation, 1 mL of 0.5 M calcium chloride solution and 4 mL of 0.5 M sodium hydroxide were added to disrupt and stop the reaction followed by filtration with Whitman's filter paper. The absorbance was determined at 410 nm using UV–Vis spectrophotometer. A unit of phosphatase enzyme activity was defined as the quantity of enzyme that was able to release 1 nmol of *p*-nitrophenol from disodium *p*-nitrophenyl phosphate in a minute, per one milligram [33]. MUB was prepared according to Tabatabai and Bremner [34]. It consisted of 3.025 g Tris-(hydroxymethyl)-aminomethane, 2.9 g maleic acid, 3.5 g citric acid, 1.57 g boric acid, 1M sodium hydroxide (NaOH) solution (122 mL), and distilled water added to a final volume of 250 mL.

### 2.6. Isolation of Genomic DNA, PCR Amplification, and Sequencing

Culture cells were harvested from a 48 hour (OD_600_ = 0.8) actively growing in a nutrient broth of NBRIB. Approximately 1.5 *μ*L (10^8^ CFU Ml^−1^) of bacterial culture were pipetted into 2 mL microtubes followed by spinning at 20,000 × *g* for 5 minutes in a centrifuge. The total DNA of selected PSB isolates was extracted using QIAmp DNA kit (Qiagen, Hilden, Germany) according to the manufacturer's protocol. The template DNA (8 *μ*l) was qualitatively checked by Gel-electrophoresis in a 1.5% agarose gel (prestained with ethidium bromide 0.5 *μ*g mL^−1^), then visualized on a UV trans-illuminator and photographed. The DNA was stored at −20°C for downstream processes.

16S ribosomal RNA gene was amplified using the following universal primers: 27f (5′AGAGTTTGATCCTGGCTCAG 3′) and 1492r (5′ TACGGCTACCTTGTTACGACTT 3′).Gene amplification was carried out in 25 *μ*L reaction volumes containing 2.5 *μ*L 10X DreamTaq buffer (100 mM Tris-HCl, pH 8.0, 500 mM KCl, and 1.5 *μ*L 25 mM MgCl), 2.0 *μ*L, 2.5 mM, dNTPs, 0.5 *μ*L of 27f primer (200 ng/*μ*L), 0.5 *μ*L of 1492r primer (200 ng/*μ*L), 0.25 *μ*L DreamTaq DNA polymerase (5U/l), and 10 *μ*l of extracted template of phosphorus solubilizing bacterial DNA. The reaction volume was accustomed to up to 25 *μ*L with sterile distilled water. The PCR thermal cycling process consisted of an initial DNA denaturation stage at 94°C for 3 minutes, followed by 35 cycles of DNA denaturation (1 min at 94°C), an annealing stage for 1 minute at 57°C, and an extension period for 2 minutes at 72°C, followed by a final elongation stay at 72°C for 8 minutes [35].

### 2.7. Molecular Phylogenetic Analysis of the PSB Isolates

The forward and reverse nucleotide contigs were merged using BioEdit 7.2 to reconstruct the full 16S rRNA genes, and aligned with CLUSTAL W. The phylogenetic tree, which contains PSB sequences of 16S rRNA gene and sequences with high similarity scores from the GenBank database, was constructed with MEGA 11.0 using the neighbor-joining method [36] with 1000 bootstrap analysis. Sequences (Supplementary Materials available here) were searched against the nonredundant nucleotide BLAST database for microbial identity. The sequences were then submitted to the NCBI GenBank database, and accession numbers were allocated as follows: ON931237, ON931235, ON931236, ON931234, ON931238, ON931233, and ON931239.

### 2.8. Determination of Potential Isolates on Improvement of Plant Biomass

We carried out an experiment in a screen house to determine the mineralization potential and phenotypic characteristics of selected isolates. Common bean varieties from Kenya Seed Company (Rose coco and Mwitemania) were used as test crops for total plant biomass. This was carried out by inoculating two high-potential PSB (KB5 and KV1) into two varieties of common bean to determine their efficacy. Certified bean seeds were surface sterilized with 1% mercuric chloride for 3 minutes followed by rinsing with distilled water and pregermination in a darkroom using Petri dishes. Inoculants were prepared according to [26]. The isolates were grown in NBRIB broth for 2 days and cells were harvested by centrifugation at 5000 × *g* for 20 min. The cells were resuspended with sterile distilled water to give a final concentration (10^8^ CFU ml^−1^) in a 250 mL conical flask. The seedlings' roots were immersed into the culture for 5 minutes and covered uniformly with a 15 mm thick layer of vermiculite in a Leonard's Jar and then placed into a completely randomized design alongside negative control (uninoculated seedlings). A total of six treatments were replicated four times to obtain 24 experimental units with two trials. Leonard's jars assemblies [37] (9 cm diameter and 12 cm height) were filled with the sterile vermiculite (Kenworks, Nairobi, Kenya). Tricalcium phosphate was provided as soil inorganic phosphorus fertilizer at the rate of 150 mg/kg based on the nutrient necessities of common bean plants [38]. Depth (5 cm) was dug into Leonard's Jar, and two seedlings were placed at equal distances. A modified nutrient solution without phosphorus was supplied to all treatments [39]. After 6 weeks, plant samples were uprooted and oven-dried at 70°C to a constant weight and were grinded after drying to determine the total dry weight in grams.

### 2.9. Statistical Data Analysis

Data were tested for homogeneity using Shapiro–Wilk and we performed a two-way analysis of variance to test the significance of isolates on varieties of common bean biomass and Pearson's correlation using the statsmodel package in python 3 to test the relationship between mineralization potential. Graphical data were plotted by the Matplotlib package in python.

## 3. Results

### 3.1. Quantitative Screening of Phosphate Mineralization by PSB Strains in Agar Plates

**Figure 2:**
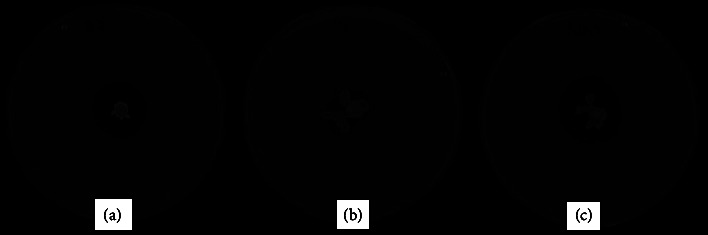
Formation of clear zones of solubilization by isolates (a) B5, (b) KB2, and (c) KB3 on an agar plate.

Formation of clear zones around the colony was an indicator of tricalcium phosphate solubilization by the isolates (Figure 2). The phosphate solubilization index of tested bacterial strains ranged from 2.3 to 4.1(Table 1). Isolate B5 displayed the highest solubilizing index of 4.17 followed by strain KV1 with 3.64. Isolate KK3 followed with 2.60, KKI with 2.54, KB3 with 2.52, and KB2 with SI 2.40. The least performed isolate was KBU with an SI of 2.34 in the agar plate.

### 3.2. Quantitative Screening of Phosphates Solubilized by Isolates in Broth Medium

**Figure 3:**
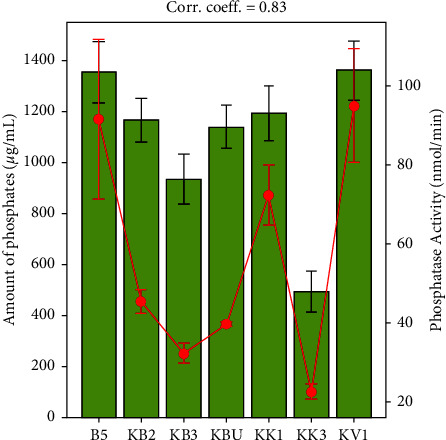
Amount of solubilized phosphorus in *μ*g/mL and phosphatase enzyme activity in nmol/min.

Isolate KV1 solubilized more phosphates in the media (1440.92 ± 92 *μ*g/mL), while isolate B5 had the potential to solubilize P of 1370.06 ± 39 *μ*g/mL (Figure 3). The isolates KK1 and KBU both solubilized P at levels of 1292.88 ± 6 *μ*g/mL and 1236.65 ± 52 *μ*g/mL, respectively, demonstrating similar phosphorus solubilization potential. Isolates KB2 and KB3, respectively, produced phosphate concentrations of 1189.03 ± 9 *μ*g/mL and 1149.15 ± 4 *μ*g/mL, and they both performed relatively similar in phosphate mineralization on agar plates. The Kakamega County KK3 isolate had the lowest solubilization potential of phosphorus in broth media (453.90 ± 36 *μ*g/mL). Each isolate was replicated thrice and data analysis revealed a significant difference (*p* < 0.05). In terms of phosphatase enzyme activity, isolate KV1 showed the highest activity at 94.92 ± 24.8 nmol/min followed by B5 (91.49 ± 34.8 nmol/min), KK1 (72.24 ± 13 nmol/min), and KB2 (45.36 ± 4.08 nmol/min), while KBU and KB3 had 39.59 ± 0.8 nmol/min and 32.22 ± 4.3 nmol/min, respectively, phosphatase activity. The least performing isolate had KK3 with 22.55 ± 3.4 nmol/min activity. Phosphatase enzyme activity strongly correlates with the amount of phosphates (correlation coefficient of *r*^2^ = 0.83) present in the medium (Figure 3).

### 3.3. Molecular Characterization and Phylogenetic Analysis of the PSB Strains

**Figure 4:**
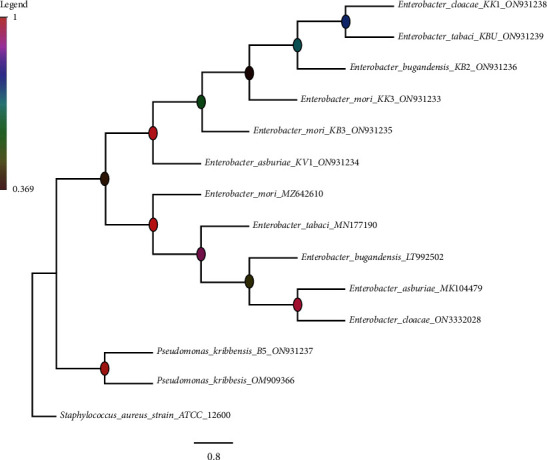
A tree showing phylogenetic relation between isolates with *Staphylococcus*_*aureus* as an outgroup. The nodes are colored per the legend in which the color corresponds to the approximate bootstrap support value.

BLAST search revealed that the isolates belong to two genera *Enterobacter* and *Pseudomonas.* Isolate B5 from Bungoma County was closely related to *Pseudomonas kribbensis* (99.60% identity), while KB2 from the same region was 98.57% relative to *Enterobacter bugandensis.* Isolate KBU which was from Busia County was identical to *Enterobacter tabaci* (99.28%), while KB3 and KK3 from Bungoma and Kakamega counties were identical to *Enterobacter mori* (99.07% and 98.51%, respectively). Isolate KVI from Vihiga county was identical to *Enterobacter asburiae*, with 98.36% identity, while KK1 from Kakamega was closely related to *Enterobacter cloacae* (98.97%) (Table 2). The consensus phylogenetic tree with bootstrap support greater than 60% is shown in Figure 4.

### 3.4. Assessment of Plant Biomass Inoculated with B5 and KV1 Isolates

**Figure 5:**
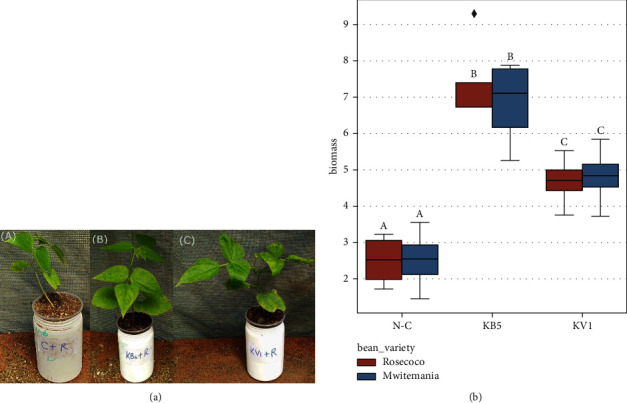
(a) Box plot showing the effects of bacteria strains inoculation on phenotypic characteristics of a Rose coco variety under phosphorus-free nutrient in a screen house: (A) noninoculated control, (B) inoculated with KB5 strain,and (C) inoculated with KV1 strain, and (b) Effects of bacteria strains on total dry weights in grams for both varieties: (A) biomass of negative control, (B) biomass of plants inoculated with KB5, and (C) biomass of plants inoculated with KV1.

The performance of the two most promising phosphate solubilizing isolates (B5 and KV1) was investigated in two common bean varieties (Rose coco and Mwitemania), commonly grown in Western Kenya [40]. All the two isolates significantly promoted the plant biomass of the two bean varieties when compared to the untreated controls (Figure 5). In terms of shoot dry weight, B5 isolate performed better in the two bean varieties as it yielded an average of 6.52 ± 1.2 grams per plant in Rose coco and 6.15 ± 1.1 grams per plant in Mwitemania. KV1 isolate yielded a shoot dry weight of 4.08 ± 0.7 grams in the Mwitemania variety and 3.97 ± 0.8 grams in the Rose coco variety. The negative controls of Mwitemania and Rose coco yielded 2.15 ± 0.8 grams and 2.06 ± 0.7 grams, respectively. In root biomass, the performance was consistently similar to shoot biomass as B5 isolate also performed greatly in both Mwitemamia and Rose coco with 0.69 ± 0.1 grams and 0.84 ± 0.1 grams, respectively. KV1 isolate followed with 0.73 ± 0.3 grams in Mwitemania and 0.72 ± 0.1 grams in Rose coco. Negative controls yielded 0.44 ± 0.1 grams in Rose coco and 0.37 ± 0.1 grams in Mwitemania. We performed a two-way ANOVA to determine if different bean varieties affected microbial efficacy, and we found that it does not (*p* > 0.05), while the two different bacterial strains affected the yield (*p* < 0.05).

## 4. Discussion

Despite the presence of nitrogen-fixing and nodulating bacteria in the roots and rhizosphere of common beans, there are other beneficial rhizobacteria including PSB that successfully colonize bean roots and nodules which also contribute to plant growth and development [41]. The early phases of plant development require soil phosphorus, which is regarded as the second most significant indication of soil fertility after nitrogen. Due to their ability to fix nitrogen through nodulation and their symbiotic association with PSB, legumes such as common beans have high P requirements [42]. Here, we show that the rhizosphere of common bean is a natural habitat for PSB that works symbiotically with nitrogen-fixing bacteria to affect the plant performance in phosphorus-depleted soils [43].

The molecular analysis of the seven isolates through partial sequencing of a 16S ribosomal gene revealed that the phosphorus solubilizing bacteria isolated from common bean rhizosphere in Western Kenya were of two generic clusters of *Enterobacter* sp. and *Pseudomonas* sp. and that they have also been extensively studied in other host plants [44, 45]. The cluster of *Enterobacter* sp. dominated the strains of study since out of the seven isolates, six were *Enterobacter*. The *Enterobacter* sp. has been previously reported in other plant rhizospheres and they have a high potential for phosphorus solubilization but very little information is associated with common beans [14, 46]. In previous studies, *Pseudomonas* sp. has been isolated and identified as one of the most efficient phosphorus solubilizing bacteria in both in monocots and dicots [12, 47, 48]. Out of the seven isolated strains from Western Kenya, two strains were assessed (B5 and KV1) for their efficacy *in vitro* and *in vivo* in the mineralization of inorganic phosphates and plant growth characteristics. Among the tested PSB strains from the region, B5 which was closely related to *Pseudomonas kribbensis* and KV1 which was closely related to *Enterobacter asburiae* displayed maximum phosphate solubilization in both agar and broth medium, respectively. These two PSB isolates showed almost a consistent and nearly equal level of phosphate solubilization in broth assay and agar assay as well as phosphatase enzyme activity. This shows that the inclination of phosphate solubilization by PSB isolates in both agar and broth assays was following a similar trend as pointed out by other studies [49–51]. The highest amount of phosphate solubilization, the maximum phosphatase enzyme activity, and the maximum potential in plant biomass recorded by isolate KV1 and isolate B5 show the future potential for bioinoculant development for sustainable agricultural production [4]. A high correlation between phosphatase activity and the amount of solubilized phosphorus is evidence that phosphatase enzyme contributes to the mechanism of phosphate solubilization ability in bacteria as previously studied [32, 52, 53].

As per the greatest performances in phosphatase, the amount of phosphates converted in agar and broth assays, B5 and KVI isolates were selected for evaluation in the screen house for their effectiveness in phenotypic characteristics of Rose coco and Mwitemania bean varieties that are mostly grown in Western Kenya. In the determination of plant biomass of the two varieties, a significant difference was noted in the isolates' performance in terms of total dry weights. Strain B5 significantly influenced the plant biomass as compared to the KV1 isolate and the negative control. B5–*Pseudomonas kribbensis* are genetically related to other previously studied *Pseudomonas* sp. including *Pseudomonas fluorescens* [45, 54] and *Pseudomonas koreensis* [55] that have been reported to highly solubilize phosphorus and promoted plant growth characteristics and therefore we recommend that the isolate may exert a vital impact in common bean nutrition, through the absorption of soluble phosphorus. Given that the KV1 strain (*Enterobacter asburiae*) has been previously reported to boost plant growth parameters under harsh conditions [13], we also report that it can boost the growth and development of leguminous plants in phosphorus-depleted soils in the current study.

Studying the impact of genetically diverse phosphorus solubilizing bacteria on the phenotypic characteristics of Rose coco and Mwitemania bean varieties, as well as assessing their mineralization potential is a way of unraveling the growth-promoting properties of these bacteria and a proof for useful bioinoculant application to leguminous crops for sustainable production in tropics [4].

## 5. Conclusion

PSB isolated from common bean rhizosphere are beneficial and can belong to a wide range of microbial communities. To the best of our understanding and literature search, the present study reported the first isolation, identification, and molecular characterization of PSB strains from the rhizosphere of common beans growing in Western Kenya soils. Among the isolated strains, we have characterized and tested two potential PSB strains KVI-*Enterobacter asburiae* and B5-*Pseudomonas kribbensis* as promising and high-efficiency strains that can be used to unravel the insufficiency of phosphorus in soil for sustainable crop production. In future studies, we recommend the use of aluminum phosphate (AIPO_4_) or iron phosphate (FePO_4_) together with tricalcium phosphate (TCP) for testing PSB. Since it is known that PSBs increase plant growth by increasing the P availability, we also recommend that the P content of the soil and plants at end of every study be measured. PSB strains colonizing common bean roots and nodules can be studied based on phosphorus activating genes, genome based variations, and metagenomics to understand the influence of genetic factors and functional mechanism on the strains and richness of endophytic microbial communities to completely assess the application of these potential strains as microbial fertilizers.

## Figures and Tables

**Table 1:** The mineralization potentials of each isolate.

Isolate	C.D	H.D	S.I
B5	0.53 ± 0.06	1.68 ± 0.10	4.17_**a**_
KB3	0.77 ± 0.15	1.17 ± 0.15	2.52_**c**_
KB2	0.93 ± 0.06	1.30 ± 0.10	2.40_**c**_
KV1	0.58 ± 0.19	1.53 ± 0.15	3.64_**ab**_
KK1	0.67 ± 0.08	1.03 ± 0.15	2.54_**c**_
KK3	0.47 ± 0.15	0.75 ± 0.12	2.60_**bc**_
KBU	0.88 ± 0.12	1.18 ± 0.16	2.34_**d**_

*p* *≤* 0.05 column C.D is colony diameter (cm), column H.D is halo zone diameter (cm), and column S.I is solubilizing index. Superscript letters indicate statistical significance.

**Table 2:** Isolates and their related BLAST strains basing on partial sequencing of 16S ribosomal gene.

Isolate ID. No.	Isolation site	NCBI P.I (%)	Strain name	Accession number
B5	Bungoma	99.60	*Pseudomonas kribbensis*	ON931237
KB3	Bungoma	99.07	*Enterobacter mori*	ON931235
KB2	Bungoma	98.57	*Enterobacter bugandensis*	ON931236
KV1	Vihiga	98.36	*Enterobacter asburiae*	ON931234
KK1	Kakamega	98.97	*Enterobacter cloacae*	ON931238
KK3	Kakamega	98.51	*Enterobacter mori*	ON931233
KBU	Busia	99.28	*Enterobacter tabaci*	ON931239

## Data Availability

The molecular datasets generated during the current study are available in the NCBI GenBank, with accession numbers: ON931237, ON931235, ON931236, ON931234, ON931238, ON931233, and ON931239. Other datasets used during the current study are available from the corresponding author upon reasonable request.

## Supplementary Materials

Supplementary Materials The 16S rRNA nucleotide sequences of the isolated bacterial species are available here.
