# Supplementary material for: Molecular Characterization and Mineralizing Potential of Phosphorus Solubilizing Bacteria Colonizing Common Bean (Phaseolus vulgaris L.) Rhizosphere in Western Kenya
Source: Int J Microbiol. 2023 Mar 1;2023:6668097. doi: 10.1155/2023/6668097 (PMC9995209; doi:10.1155/2023/6668097)
Supplement: Supplementary Materials — The 16S rRNA nucleotide sequences of the isolated bacterial species are available here. [file 6668097.f1.docx]

**16S rRNA nucleotide sequences of the isolated bacterial species**

>KK3

CTGATTACGCGATTCCGACTTCATGGAGTCGAGTTGCAGACTCCAATCCGGACTACGACG

CACTTTATGAGGTCCGCTTGCTCTCGCGAGGTCGCTTCTCTTTGTATGCGCCATTGTAGC

ACGTGTGTAGCCCTACTCGTAAGGGCCATGATGACTTGACGTCATCCCCACCTTCCTCCA

GTTTATCACTGGCAGTCTCCTTTGAGTTCCCGGCCACCGCTGGCAACAAAGGATAAGGGT

TGCGCTCGTTGCGGGACTTAACCCAACATTTCACAACACGAGCTGACGACAGCCATGCAG

CACCTGTCTCAGAGTTCCCGAAGGCACCAATCCATCTCTGGAAAGTTCTCTGGATGTCAA

GAGTAGGTAAGGTTCTTCGCGTTGCATCGAATTAAACCACATGCTCCACCGCTTGTGCGG

GCCCCCGTCAATTCATTTGAGTTTTAACCTTGCGGCCGTACTCCCCAGGCGGTCGACTTA

ACGCGTTAGCTCCGGAAGCCACGCCTCAAGGGCACAACCTCCAAGTCGACATCGTTTACG

GCGTGGACTACCAGGGTATCTAATCCTGTTTGCTCCCCACGCTTTCGCACCTGAGCGTCA

GTCTTTGTCCAGGGGGCCGCCTTCGCCACCGGTATTCCTCCAGATCTCTACGCATTTCAC

CGCTACACCTGGAATTCTACCCCCCTCTACAAGACTCTAGCCTGCCAGTTTCGAATGCAG

TTCCCAGGTTGAGCCCGGGGATTTCACATCCGACTTGACAGACCGCCTGCGTGCGCTTTA

CGCCCAGTAATTCCGATTAACGCTTGCACCCTCCGTATTACCGCGGCTGCTGGCACGGAG

TTAGCCGGTGCTTCTTCTGCGGGTAACGTCAAGGGTTATTAACCTATCCCTTCCTCCCCG

CTGAAAGTACTTTACAACCCGAAGGCCTTCTTCATACACGCGGCATGGCTGCATCAGGCT

TGCGCCCATTGTGCAATATTCCCCACTGCTGCCTCCCGTAGGAGTCTGGACCGTGTCTCA

GTTCCAGTGCTGGTCATCCTCTCAACCAGCTAGGGATCGTCA

>KV1

TAAGCGATTCCGACTTCATGGAGTCGAGTTGCAGACTCCAATCCGGACTACGACGCACTT

TATGAGGTCCGCTTGCTCTCGCGAGGTCGCTTCTCTTTGTATGCGCCATTGTAGCACGTG

TGTAGCCCTACTCGTAAGGGCCATGATGACTTGACGTCATCCCCACCTTCCTCCAGTTTA

TCACTGGCAGTCTCCTTTGAGTTCCCGGCACCGCTGGCAACAAAGGATAAGGGTTGCGCT

CGTTGCGGGACTTAACCCAACATTTCACAACACGAGCTGACGACAGCCATGCAGCACCTG

TCTCAGAGTTCCCGAAGGCACCAATCCATCTCTGGAAAGTTCTCTGGATGTCAAGAGTAG

GTAAGGTTCTTCGCGTTGCATCGAATTAAACCACATGCTCCACCGCTTGTGCGGGCCCCC

GTCAATTCATTTGAGTTTTAACCTTGCGGCCGTACTCCCCAGGCGGTCGACTTAACGCGT

TAGCTCCGGAAGCCACGCCTCAAGGGCACAACCTCCAAGTCGACATCGTTTACGGCGTGG

ACTACCAGGGTATCTAATCCTGTTTGCTCCCCACGCTTTCGCACCTGAGCGTCAGTCTTT

GTCCAGGGGGCCGCCTTCGCCACCGGTATTCCTCCAGATCTCTACGCATTTCACCGCTAC

ACCTGGAATTCTACCCCCCTCTACAAGACTCTAGCCTGCCAGTTTCGAATGCAGTTCCCA

GGTTGAGCCCGGGGATTTCACATCCGACTTGACAGACCGCCTGCGTGCGCTTTACGCCCA

GTAATTCCGATTAACGCTTGCACCCTCCGTATTACCGCGGCTGCTGGCACGGAGTTAGCC

GGTGCTTCTTCTGCGGGTAACGTCAAGGGTTATTAACCTTCCCTTCCTCCCCGCTGAAAG

TACTTTACAACCCGAAGGCCTTCTTCATACACGCGGCATGGCTGCATCAGGCTTGCGCCC

ATTGTGCATATTCCCCACTGCTGCCTCCCGTAGGCTGGACCGTGTCTCAGTTCCAGTGCT

GGCATCCTC

>KB3

CTTACTAGCGATTCCGACTTCATGGAGTCGAGTTGCAGACTCCAATCCGGACTACGACGC

ACTTTATGAGGTCCGCTTGCTCTCGCGAGGTCGCTTCTCTTTGTATGCGCCATTGTAGCA

CGTGTGTAGCCCTACTCGTAAGGGCCATGATGACTTGACGTCATCCCCACCTTCCTCCAG

TTTATCACTGGCAGTCTCCTTTGAGTTCCCGCCGAACCGCTGGCAACAAAGGATAAGGGT

TGCGCTCGTTGCGGGACTTAACCCAACATTTCACAACACGAGCTGACGACAGCCATGCAG

CACCTGTCTCAGAGTTCCCGAAGGCACCAATCCATCTCTGGAAAGTTCTCTGGATGTCAA

GAGTAGGTAAGGTTCTTCGCGTTGCATCGAATTAAACCACATGCTCCACCGCTTGTGCGG

GCCCCCGTCAATTCATTTGAGTTTTAACCTTGCGGCCGTACTCCCCAGGCGGTCGACTTA

ACGCGTTAGCTCCGGAAGCCACGCCTCAAGGGCACAACCTCCAAGTCGACATCGTTTACG

GCGTGGACTACCAGGGTATCTAATCCTGTTTGCTCCCCACGCTTTCGCACCTGAGCGTCA

GTCTTTGTCCAGGGGGCCGCCTTCGCCACCGGTATTCCTCCAGATCTCTACGCATTTCAC

CGCTACACCTGGAATTCTACCCCCCTCTACAAGACTCTAGCCTGCCAGTTTCGAATGCAG

TTCCCAGGTTGAGCCCGGGGATTTCACATCCGACTTGACAGACCGCCTGCGTGCGCTTTA

CGCCCAGTAATTCCGATTAACGCTTGCACCCTCCGTATTACCGCGGCTGCTGGCACGGAG

TTAGCCGGTGCTTCTTCTGCGGGTAACGTCAATTGCTGAGGTTATTAACCTCAACACCTT

CCTCCCCGCTGAAAGTACTTTACAACCCGAAGGCCTTCTTCATACACGCGGCATGGCTGC

ATCAGGCTTGCGCCCATTGTGCAATATTCCCCACTGCTGCCTCCCGTAGGAGTCTGGACC

GTGTCTCAGTTCCAGTGCTGCATCCTCTCCAGCTAGGGATCGTCT

>KB2

GTTACAGCGATTCCGACTTCATGGAGTCGAGTTGCAGACTCCAATCCGGACTACGACGCA

CTTTATGAGGTCCGCTTGCTCTCGCGAGGTCGCTTCTCTTTGTATGCGCCATTGTAGCAC

GTGTGTAGCCCTACTCGTAAGGGCCATGATGACTTGACGTCATCCCCACCTTCCTCCAGT

TTATCACTGGCAGTCTCCTTTGAGTTCCCGCCGAACGCTGGCAACAAAGGATAAGGGTTG

CGCTCGTTGCGGGACTTAACCCAACATTTCACAACACGAGCTGACGACAGCCATGCAGCA

CCTGTCTCAGAGTTCCCGAAGGCACCAATCCATCTCTGGAAAGTTCTCTGGATGTCAAGA

GTAGGTAAGGTTCTTCGCGTTGCATCGAATTAAACCACATGCTCCACCGCTTGTGCGGGC

CCCCGTCAATTCATTTGAGTTTTAACCTTGCGGCCGTACTCCCCAGGCGGTCGACTTAAC

GCGTTAGCTCCGGAAGCCACGCCTCAAGGGCACAACCTCCAAGTCGACATCGTTTACGGC

GTGGACTACCAGGGTATCTAATCCTGTTTGCTCCCCACGCTTTCGCACCTGAGCGTCAGT

CTTTGTCCAGGGGGCCGCCTTCGCCACCGGTATTCCTCCAGATCTCTACGCATTTCACCG

CTACACCTGGAATTCTACCCCCCTCTACAAGACTCTAGCCTGCCAGTTTCGAATGCAGTT

CCCAGGTTGAGCCCGGGGATTTCACATCCGACTTGACAGACCGCCTGCGTGCGCTTTACG

CCCAGTAATTCCGATTAACGCTTGCACCCTCCGTATTACCGCGGCTGCTGGCACGGAGTT

AGCCGGTGCTTCTTCTGCGGGTAACGTCAATTGCTGAGGTTATTAACCTCAACACCTTCC

TCCCCGCTGAAAGTACTTTACAACCCGAAGGCCTTCTTCATACACGCGGCATGGCTGCAT

CAGGCTTGCGCCCATTGTGCAATATTCCCCACTGCTGCCTCCCGTAGGAGTCTGGACCGT

GTCTCAGTTCCAGTGGCTGGTCATCCTCTACCAGCTAGGGATCGTCTAGGTGAGCCGTTA

CCCCTACTAGCTAATCCATCTGGG

>B5

CCGGCGGGGTGAGTAATGCCTAGGAATCTGCCTGGTAGTGGGGGACAACGTTTCGAAAGG

AACGCTAATACCGCATACGTCCTACGGGAGAAAGCAGGGGACCTTCGGGCCTTGCGCTAT

CAGATGAGCCTAGGTCGGATTAGCTAGTTGGTGAGGTAATGGCTCACCAAGGCGACGATC

CGTAACTGGTCTGAGAGGATGATCAGTCACACTGGAACTGAGACACGGTCCAGACTCCTA

CGGGAGGCAGCAGTGGGGAATATTGGACAATGGGCGAAAGCCTGATCCAGCCATGCCGCG

TGTGTGAAGAAGGTCTTCGGATTGTAAAGCACTTTAAGTTGGGAGGAAGGGTTGTAGATT

AATACTCTGCAATTTTGACGTTACCGACAGAATAAGCACCGGCTAACTCTGTGCCAGCAG

CCGCGGTAATACAGAGGGTGCAAGCGTTAATCGGAATTACTGGGCGTAAAGCGCGCGTAG

GTGGTTCGTTAAGTTGGATGTGAAATCCCCGGGCTCAACCTGGGAACTGCATCCAAAACT

GGCGAGCTAGAGTATGGTAGAGGGTGGTGGAATTTCCTGTGTAGCGGTGAAATGCGTAGA

TATAGGAAGGAACACCAGTGGCGAAGGCGACCACCTGGACTGATACTGACACTGAGGTGC

GAAAGCGTGGGGAGCAAACAGGATTAGATACCCTGGTAGTCCACGCCGTAAACGATGTCA

ACTAGCCGTTGGGAGCCTTGAGCTCTTAGTGGCGCAGCTAACGCATTAAGTTGACCGCCT

GGGGAGTACGGCCGCAAGGTTAAAACTCAAATGAATTGACGGGGGCCCGCACAAGCGGTG

GAGCATGTGGTTTAATTCGAAGCAACGCGAAGAACCTTACCAGGCCTTGACATCCAATGA

ACTTTCCAGAGATGGATTGGTGCCTTCGGGAACATTGAGACAGGTGCTGCATGGCTGTCG

TCAGCTCGTGTCGTGAGATGTTGGGTTAAGTCCCGTAACGAGCGCAACCCTTGTCCTTAG

TTACCAGCACGTTATGGTGGGCACTCTAAGGAGACTGCCGGTGACAAACCGGAGGAAGGT

GGGGATGACGTCAAGTCATCATGGCCCTTACGGCCTGGGCTACACACGTGCTACAATGGT

CGGTACAAAGGGTTGCCAAGCCGCGAGGTGGAGCTAATCCCATAAAACCGATCGTAGTCC

GGATCGCAGTCTGCAACTCGACTGCGTGAAGTCGGAATCGCTTAATCCG

>KK1

GTTACTAGCGATTCCGACTTCATGGAGTCGAGTTGCAGACTCCAATCCGGACTACGACGC

ACTTTATGAGGTCCGCTTGCTCTCGCGAGGTCGCTTCTCTTTGTATGCGCCATTGTAGCA

CGTGTGTAGCCCTACTCGTAAGGGCCATGATGACTTGACGTCATCCCCACCTTCCTCCAG

TTTATCACTGGCAGTCTCCTTTGAGTTCCCGGCCACCGCTGGCAACAAAGGATAAGGGTT

GCGCTCGTTGCGGGACTTAACCCAACATTTCACAACACGAGCTGACGACAGCCATGCAGC

ACCTGTCTCAGAGTTCCCGAAGGCACCAAAGCATCTCTGCTAAGTTCTCTGGATGTCAAG

AGTAGGTAAGGTTCTTCGCGTTGCATCGAATTAAACCACATGCTCCACCGCTTGTGCGGG

CCCCCGTCAATTCATTTGAGTTTTAACCTTGCGGCCGTACTCCCCAGGCGGTCGACTTAA

CGCGTTAGCTCCGGAAGCCACGCCTCAAGGGCACAACCTCCAAGTCGACATCGTTTACGG

CGTGGACTACCAGGGTATCTAATCCTGTTTGCTCCCCACGCTTTCGCACCTGAGCGTCAG

TCTTTGTCCAGGGGGCCGCCTTCGCCACCGGTATTCCTCCAGATCTCTACGCATTTCACC

GCTACACCTGGAATTCTACCCCCCTCTACAAGACTCTAGCCTGCCAGTTTCGAATGCAGT

TCCCAGGTTGAGCCCGGGGATTTCACATCCGACTTGACAGACCGCCTGCGTGCGCTTTAC

GCCCAGTAATTCCGATTAACGCTTGCACCCTCCGTATTACCGCGGCTGCTGGCACGGAGT

TAGCCGGTGCTTCTTCTGCGGGTAACGTCAATCGACAGGGTTATTAACCCTGTCGCCTTC

CTCCCCGCTGAAAGTACTTTACAACCCGAAGGCCTTCTTCATACACGCGGCATGGCTGCA

TCAGGCTTGCGCCCATTGTGCAATATTCCCCACTGCTGCCTCCCGTAGGAGTCTGGACCG

TGTCTCAGTTCCAGTGTGGCTGGTCATCCTCTCAGACCAGCTAGGGATCGTCGCCTAGGT

GAGCCGTTACCCCACCTACTAGCTAATCCCATCTGGGCACATCCGATGGCAAGAGGCCCG

AAGGTCCCCCTCTTTGGTCTTGCGACGTTATGCGGTATTAGCTACCGTTTCCAGTAGTTA

TCCCCCTCCATCAGGCACCCAGACATTACTCACCCCCGCCACTCCAGCGAAGCAGCAAGT

CT

>KBU

ATACGATTACAGCGATTCCGACTTCATGGGAGTCGAGTTGCAGACTCCAATCCGGACTAC

GACGCACTTTATGAGGTCCGCTTGCTCTCGCGAGGTCGCTTCTCTTTGTATGCGCCATTG

TAGCACGTGTGTAGCCCTACTCGTAAGGGCCATGATGACTTGACGTCATCCCCACCTTCC

TCCAGTTTATCACTGGCAGTCTCCTTTGAGTTCCCGGCCTAACCGCTGGCAACAAAGGAT

AAGGGTTGCGCTCGTTGCGGGACTTAACCCAACATTTCACAACAMGAGCTGACGACAGCC

ATGCAGCACCTGTCTCAGAGTTCCCGAAGGCACCAATCCATCTCTGGAAAGTTCTCTGGA

TGTCAAGAGTAGGTAAGGTTCTTCGCGTTGCATCGAATTAAACCACATGCTCCACCGCTT

GTGCGGGCCCCCGTCAATTCATTTGAGTTTTAACCTTGCGGCCGTACTCCCCAGGCGGTC

GACTTAACGCGTTAGCTCCGGAAGCCACGCCTCAAGGGCACAACCTCCCAAGTCGACATC

GTTTACGGCGTGGACTACCAGGGTATCTAATCCTGTTTGCTCCCCACGCTTTCGCACCTG

AGCGTCAGTCTTTGTCCAGGGGGCCGCCTTCGCCACCGGTATTCCTCCAGATCTCTACGC

ATTTCACCGCTACACCTGGAATTCTACCCCCCTCTACAAGACTCTAGCCTGCCAGTTTCG

AATGCAGTTCCCAGGTTGAGCCCGGGGATTTCACATCCGACTTGACAGACCGCCTGCGTG

CGCTTTACGCCCAGTAATTCCGATTAACGCTTGCACCCTCCGTATTACCGCGGCTGCTGG

CACGGAGTTAGCCGGTGCTTCTTCTGCGGGTAACGTCAATTGCTGAGGTTATTAACCTCA

GCACCTTCCTCCCCGCTGAAAGTACTTTACAACCCGAAGGCCTTCTTCATACACGCGGCA

TGGCTGCATCAGGCTTGCGCCCATTGTGCAATATTCCCCACTGCTGCCTCCCGTAGGAGT

CTGGACCGTGTCTCAGTTCCAGTGTGGCTGGTCATCCTCTCAGACCAGCTAGGGATCGTC

GCCTAGGTGAGCCGTTACCCCACCTACTAGCTAATCCCATCTGGGCACATCTGATGGCAA

GAGGCCCGAAGGTCCCCCTCTTTGGTCTTGCGACGTTATGCGGTATTAGCTACCGTTTCC

AGTAGTTATCCCCCTCCATCAGGCAGTTCCCAGACATTACTCACCCTCCGCCGCTCCACC
